# Editorial: Differentiating and understanding the multifaceted nature of Traumatic Brain Injury using clinical and pre-clinical systems

**DOI:** 10.3389/fneur.2026.1827663

**Published:** 2026-04-08

**Authors:** Aarti Gautam, Venkatasivasai Sujith Sajja, Richard S. Lee, Ruoting Yang, Rasha Hammamieh

**Affiliations:** 1 Walter Reed Army Institute of Research, Silver Spring, MD, United States; 2 The Geneva Foundation, Tacoma, WA, United States; 3 Department of Psychiatry and Behavioral Science, John Hopkins University, Baltimore, MD, United States

**Keywords:** biomarkers, network biology, neuroinflammation, translational validation, Traumatic Brain Injury

Traumatic Brain Injury (TBI) remains a formidable obstacle in neurology, primarily due to the vast biological variance in how it manifests. No two injuries are identical; each is dictated by specific biomechanical forces, acute physiological reactions, and the patient's own genetic, epigenetic and health background. While clinical practice has traditionally relied on a broad scale from mild concussion to severe trauma, these labels often fail to account for the shifting cellular environment occurring in the hours following an impact. This Research Topic assembles a body of work intended to align experimental findings with clinical needs by prioritizing a deep, data-driven look at the brain's internal networks.

The overarching premise here is that TBI cannot be understood by examining variables in isolation. By anchoring clinical observations in high-fidelity experimental models, the contributors have mapped the molecular signaling cascades that determine the trajectory toward either neuro-regeneration or chronic decline. Rather than tracing a single pathway, these studies emphasize the intersections of dysfunction—those specific intersections where neuroinflammation, metabolic exhaustion, and structural degradation converge.

Transitioning toward precision medicine approaches requires moving away from standardized, broad-brush protocols and toward interventions that account for an individual's specific molecular state. This is evident in the contributions investigating the “second hit,” where the initial mechanical trauma triggers an inflammatory cascade that often causes more extensive damage than the impact itself. One study tracks cytokine flux to identify signaling nodes that might be targeted to halt this progression, while another uses transcriptomic profiling to prove that mild and severe injuries are not just different in degree, but represent entirely different biological states requiring distinct management strategies.

As the volume of neurotrauma data expands, computational biology has become a cornerstone of the field. The network analyses included in this topic demonstrate how brain connectivity is forced to reorganize or collapses entirely under the weight of trauma. Furthermore, the dynamic models presented here simulate the interplay between immune response and neuronal architecture during the acute phase, offering a predictive framework that could eventually allow clinicians to anticipate a patient's recovery path before the symptoms even fully surface.

Refining our diagnostic toolkit remains a priority. Several authors explore the utility of microRNAs and extracellular vesicles in the blood as objective markers of injury. By linking these molecular signatures to specific recovery patterns, this research moves the field closer to a future where a blood draw provides a concrete prognosis and a specific plan for long-term rehabilitation.

However, the path from the bench to the clinic is rarely straightforward. This Research Topic addresses the high rate of failure in clinical trials by advocating for more rigorous models, such as repetitive mild TBI (rmTBI) protocols that mirror the cumulative nature of contact sports. There is also a strong push for the use of large animal models to provide the physiological parallels needed to validate high-stakes therapies. Crucially, the research looks beyond the sterile environment of a laboratory to account for real-world clinical confounders. By examining how an aging brain or chronic pre-existing stress alters the inflammatory response, these articles remind us that the trauma is only part of the equation; the patient's pre-existing biological context ultimately dictates the outcome. An injury sustained by an aged, stressed system does not trigger the same repair mechanisms as one in a healthy subject, and our research must account for these host-specific variables to achieve actual clinical utility.

The research highlighted here confirms that TBI is a complex network-wide disease that demands a comprehensive, multi-level response. By synthesizing multi-omics, machine learning, and refined experimental models, we are finally seeing the emergence of a precision-medicine framework that respects the underlying complexity of human neurobiology. We are grateful to the investigators and peer reviewers who contributed their expertise to this foundation for the next wave of multidisciplinary TBI research.

